# Supplementary material for: Increased Oral Care Needs and Third Molar Symptoms in Women with Gestational Diabetes Mellitus: A Finnish Gestational Diabetes Case–Control Study
Source: Int J Environ Res Public Health. 2022 Aug 28;19(17):10711. doi: 10.3390/ijerph191710711 (PMC9518339; doi:10.3390/ijerph191710711)
Supplement: Supplementary file 1 [file ijerph-19-10711-s001.zip › Supplement Questionnaire S1.pdf]

Supplement Questionnaire S1. Oral health questions and answer options in the questionnaire. One option to be chosen unless otherwise specified.

Personal health:

1. "How do you describe your oral care at the moment?":
"High", "Intermediate", "Low", "Very low", "I don't have need for oral care in my opinion" or "I cannot say".

2. "How many restored teeth do you have?":
"None", "1-4", "5-10", "Over 10" or "I cannot say".

3. "Do your gums bleed or have they bled when brushing your teeth?":
"Almost constantly", "Weekly", "Occasionally", "Never" or "I cannot say".

4. "Do you have any third molars removed?" (You may choose several options):
"No", "Yes, one from upper jaw", "Yes, both from upper jaw", "Yes, one from lower jaw", "Yes, both from lower jaw" or "I cannot say".

5. "Are your third molars causing any discomfort or pain?":
"No", "Yes" or "I cannot say".

6. "Do you grind your teeth?":
"Every night", "Weekly", "Occasionally", "Never" or "I cannot say".
