# Supplementary material for: Increased Oral Care Needs and Third Molar Symptoms in Women with Gestational Diabetes Mellitus: A Finnish Gestational Diabetes Case–Control Study
Source: Int J Environ Res Public Health. 2022 Aug 28;19(17):10711. doi: 10.3390/ijerph191710711 (PMC9518339; doi:10.3390/ijerph191710711)
Supplement: Supplementary file 1 [file ijerph-19-10711-s001.zip › Supplement Table S1.pdf]

Supplement Table S1. Associations between oral health and maternal background characteristics.

		Removed third molar (yes/no)	Number of removed third molars	Third molar symptoms	Gingival bleeding	Restored teeth (> 10)	Maternal age	Parity	Pre-pregnancy BMI
**Need for oral care**	OR	0.83	0.93	1.14	2.04	1.88	0.98	1.03	1.05
	95% CI	(0.68–1.03)	(0.88–0.99)	(0.93–1.40)	(1.72–2.42)	(1.65–2.15)	(0.96–1.00)	(1.01–1.05)	(1.00–1.10)
	P value	0.085	0.025	0.206	< 0.001	< 0.001	0.027	< 0.001	0.038
	Missing, *n* (%)	38 (1.9%)	38 (1.9%)	73 (3.7%)	35 (1.8%)	153 (7.8%)	17 (0.9%)	18 (0.9%)	17 (0.9%)
**Removed third molar (yes/no)**	OR			6.42	1.12	1.46	1.13	1.02	1.12
	95% CI			(5.02–8.21)	(0.95–1.32)	(1.30–1.66)	(1.10–1.15)	(1.00–1.04)	(1.06–1.19)
	P value			< 0.001	0.168	< 0.001	< 0.001	0.039	< 0.001
	Missing *n* (%)			76 (3.9)	42 (2.1)	165 (8.4)	23 (1.2)	24 (1.2)	23 (1.2)
**Third molar symptoms**	OR	6.42	1.48		1.11	1.00	0.99	1.01	1.00
	95% CI	(5.02–8.21)	(1.39–1.57)		(0.95–1.30)	(0.89–1.12)	(0.98–1.01)	(1.00–1.03)	(0.95–1.04)
	P value	< 0.001	< 0.001		0.181	0.954	0.451	0.082	0.832
	Missing *n* (%)	76 (3.9)	76 (3.9)		78 (4.0)	193 (9.8)	59 (3.0)	60(3.1)	59 (3.0)
**Gingival bleeding**	OR	1.12	0.96	1.11		1.62	0.95	1.01	0.93
	95% CI	(0.95–1.32)	(0.82–1.11)	(0.95–1.30)		(1.18–2.22)	(0.90–0.99)	(0.97–1.06)	(0.81–1.08)
	P value	0.168	0.562	0.181		0.003	0.017	0.566	0.367
	Missing *n* (%)	42 (2.1)	42 (2.1)	78 (4.0)		159 (8.1)	21 (1.1)	22 (1.1)	21 (1.1)
**Restored teeth**	OR	1.46	1.32	1.00	1.62		1.07	1.03	1.09
	95% CI	(1.30–1.66)	(1.20–1.45)	(0.89–1.12)	(1.18–2.22)		(1.04–1.10)	(1.00–1.05)	(1.02–1.16)
	P value *n* (%)	< 0.001	< 0.001	0.954	0.003		< 0.001	0.035	0.010

	Missing	165 (8.4)	165 (8.4)	193 (9.8)	159 (8.1)		144 (7.3)	145 (7.4)	144 (7.3)
		Maternal smoking	Educational attainment	History of asthma	History of insomnia and/or mental disorders	Chronic hypertension	Gestational hypertension or Pre-eclampsia		Hyperemesis
**Need for oral care**	OR	1.42	0.84	1.24	1.57				
	95% CI	(1.10–1.85)	(0.75–0.94)	(0.90–1.72)	(1.18–2.09)				
	P value	0.008	0.003	0.194	0.002				
	Missing *n* (%)	17 (0.9)	17 (0.9)	100 (5.1)	98 (5.0)				
**Removed third molar (yes/no)**	OR	0.81	1.31	1.18	1.28				
	95% CI	(0.63–1.04)	(1.31–1.46)	(0.85–1.63)	(0.95–1.72)				
	P value	0.091	< 0.001	0.329	0.102				
	Missing *n* (%)	23 (1.2)	23 (1.2)	103 (5.2)	103 (5.2)				
**Third molar symptoms**	OR	1.66	0.81	1.45	1.51				
	95% CI	(1.29–2.13)	(0.73–0.90)	(1.07–1.97)	(1.15–1.98)				
	P value	< 0.001	< 0.001	0.017	0.003				
	Missing *n* (%)	59 (3.0)	59 (3.0)	137 (7.0)	136 (6.9)				
**Gingival bleeding**	OR	1.55	0.60	0.92	2.08	1.24	1.20		
	95% CI	(0.86–2.78)	(0.44–0.81)	(0.39–2.16)	(1.15–3.75)	(0.60–2.53)	(0.68–2.09)		
	P value	0.144	< 0.001	0.845	0.016	0.562	0.533		
	Missing *n* (%)	21 (1.1)	21 (1.1)	103 (5.2)	102 (5.2)	22 (1.1)	22 (1.1)		
**Restored teeth**	OR	1.12	1.05	1.30	1.03				1.00
	95% CI	(0.75–1.68)	(0.89–1.23)	(0.82–2.06)	(0.66–1.61)				(0.95–1.06)
	P value	0.576	0.595	0.264	0.893				0.997
	Missing *n* (%)	144 (7.3)	144 (7.3)	219 (11.1)	217 (11.0)				157 (8.0)

BMI, body mass index; OR, odds ratio; CI, confidence interval.
Missing values in number (percentage).
The chi-square test was the chosen analytical method. “Cannot say” answers were removed from the analyses.
