# Supplementary material for: Increased Oral Care Needs and Third Molar Symptoms in Women with Gestational Diabetes Mellitus: A Finnish Gestational Diabetes Case–Control Study
Source: Int J Environ Res Public Health. 2022 Aug 28;19(17):10711. doi: 10.3390/ijerph191710711 (PMC9518339; doi:10.3390/ijerph191710711)
Supplement: Supplementary file 1 [file ijerph-19-10711-s001.zip › Supplement Table S2.pdf]

Supplement Table S2. The logistic regression analyses of oral health outcomes with (presented in main document) and without missing variables according to women's gestational diabetes mellitus (GDM) status.

							Model 1				Model 2				Model 3			
**GDM-Control**		**Total *N***	**OR**	**(95% CI)**	***n*[a]**	**(%)**	**aOR**	**(95% CI)**	***n*[a]**	**(%)**	**aOR**	**(95% CI)**	***n*[a]**	**(%)**	**aOR**	**(95% CI)**	***n*[a]**	**(%)**
Need for oral care	[b]	**1859**	1.38	(1.12-1.69)	1844	(99.2)	1.53	(1.24-1.90)	1844	(99.2)	1.39	(1.10-1.74)	1844	(99.2)	1.36	(1.08-1.72)	1844	(99.2)
	[c]	**1965**	1.39	(1.13-1.69)	1948	(99.1)	1.54	(1.25-1.89)	1948	(99.1)	1.40	(1.12-1.75)	1948	(99.1)	1.36	(1.08-1.72)	1844	(93.8)
Gingival bleeding	[b]	**1858**	1.13	(0.83-1.53)	1839	(99.0)	1.22	(0.89-1.67)	1839	(99.0)	1.19	(0.85-1.66)	1839	(99.0)	1.16	(0.82-1.63)	1839	(99.0)
	[c]	**1965**	1.09	(0.81-1.47)	1944	(98.9)	1.19	(0.87-1.61)	1944	(98.9)	1.17	(0.84-1.63)	1943	(98.9)	1.16	(0.82-1.63)	1839	(93.6)
Symptoms from the third molar	[b]	**1859**	1.16	(0.96-1.40)	1807	(97.2)	1.19	(0.98-1.45)	1807	(97.2)	1.13	(0.92-1.40)	1807	(97.2)	1.09	(0.88-1.35)	1807	(97.2)
	[c]	**1965**	1.17	(0.97-1.41)	1906	(97.0)	1.20	(0.99-1.46)	1906	(97.0)	1.14	(0.93-1.40)	1905	(96.9)	1.09	(0.88-1.34)	1807	(92.0)
Removed third molar	[b]	**1859**	1.29	(1.06-1.56)	1840	(99.0)	0.97	(0.79-1.19)	1840	(99.0)	0.94	(0.75-1.17)	1840	(99.0)	0.94	(0.75-1.17)	1840	(99.0)
	[c]	**1965**	1.27	(1.05-1.53)	1942	(98.8)	0.95	(0.78-1.16)	1942	(98.8)	0.92	(0.74-1.14)	1941	(98.8)	0.94	(0.75-1.17)	1840	(93.6)
Restored teeth	[b]	**1847**	1.43	(1.05-1.95)	1715	(92.9)	1.21	(0.88-1.68)	1715	(92.9)	1.13	(0.80-1.60)	1715	(92.9)	1.12	(0.79-1.59)	1715	(92.9)
	[c]	**1965**	1.44	(1.06-1.94)	1821	(92.7)	1.21	(0.89-1.66)	1821	(92.7)	1.14	(0.81-1.60)	1820	(92.6)	1.12	(0.79-1.59)	1715	(87.3)
**Pharmacologically treated GDM-Control**																		
Need for oral care	[b]	**1078**	1.53	(1.08-2.16)	1067	(99.0)	1.75	(1.21-2.52)	1067	(99.0)	1.60	(1.07-2.39)	1067	(99.0)	1.58	(1.05-2.39)	1067	(99.0)
	[c]	**1131**	1.48	(1.06-2.07)	1120	(99.0)	1.69	(1.19-2.42)	1120	(99.0)	1.53	(1.03-2.27)	1120	(99.0)	1.58	(1.05-2.39)	1067	(94.3)
Gingival bleeding	[b]	**1078**	1.21	(0.73-2.02)	1070	(99.3)	1.42	(0.84-2.43)	1070	(99.3)	1.57	(0.87-2.81)	1070	(99.3)	1.51	(0.83-2.74)	1070	(99.3)
	[c]	**1131**	1.12	(0.68-1.84)	1123	(99.3)	1.33	(0.78-2.25)	1123	(99.3)	1.45	(0.81-2.59)	1123	(99.3)	1.51	(0.83-2.74)	1070	(94.6)
Symptoms from the third molar	[b]	**1078**	1.63	(1.18-2.25)	1044	(96.8)	1.69	(1.20-2.37)	1044	(96.8)	1.82	(1.25-2.65)	1044	(96.8)	1.69	(1.15-2.48)	1044	(96.8)
	[c]	**1131**	1.57	(1.15-2.15)	1093	(96.6)	1.61	(1.16-2.25)	1093	(96.6)	1.73	(1.20-2.50)	1093	(96.6)	1.69	(1.15-2.48)	1044	(92.3)
Removed third molar	[b]	**1078**	1.49	(1.05-2.12)	1066	(98.9)	0.93	(0.64-1.37)	1066	(98.9)	0.88	(0.58-1.33)	1066	(98.9)	0.88	(0.58-1.34)	1066	(98.9)
	[c]	**1131**	1.38	(0.99-1.93)	1118	(98.9)	0.85	(0.59-1.22)	1118	(98.9)	0.81	(0.54-1.21)	1118	(98.9)	0.88	(0.58-1.34)	1066	(94.3)

Restored teeth	b	**1072**	1.09	(0.63-1.90)	994	(92.7)	0.82	(0.46-1.47)	994	(92.7)	0.76	(0.40-1.46)	994	(92.7)	0.76	(0.39-1.48)	994	(92.7)
	c	**1131**	1.14	(0.67-1.93)	1049	(92.7)	0.83	(0.47-1.46)	1049	(92.7)	0.76	(0.40-1.42)	1049	(92.7)	0.76	(0.39-1.48)	994	(87.9)
**Diet-treated GDM-Control**																		
Need for oral care	b	**1649**	1.34	(1.08-1.67)	1637	(99.3)	1.51	(1.20-1.89)	1637	(99.3)	1.39	(1.09-1.77)	1637	(99.3)	1.37	(1.08-1.75)	1637	(99.3)
	c	**1740**	1.36	(1.10-1.69)	1726	(99.2)	1.53	(1.23-1.91)	1726	(99.2)	1.42	(1.13-1.80)	1725	(99.1)	1.37	(1.08-1.75)	1637	(94.1)
Gingival bleeding	b	**1648**	1.09	(0.79-1.51)	1630	(98.9)	1.16	(0.83-1.62)	1630	(98.9)	1.07	(0.75-1.52)	1630	(98.9)	1.04	(0.73-1.50)	1630	(98.9)
	c	**1740**	1.06	(0.77-1.45)	1720	(98.9)	1.13	(0.82-1.57)	1720	(98.9)	1.05	(0.74-1.49)	1719	(98.8)	1.04	(0.73-1.50)	1630	(93.7)
Symptoms from the third molar	b	**1649**	1.05	(0.86-1.29)	1604	(97.3)	1.10	(0.89-1.36)	1604	(97.3)	1.04	(0.83-1.30)	1604	(97.3)	1.00	(0.80-1.26)	1604	(97.3)
	c	**1740**	1.07	(0.88-1.30)	1689	(97.1)	1.12	(0.91-1.37)	1689	(97.1)	1.06	(0.85-1.31)	1688	(97.0)	1.00	(0.80-1.25)	1604	(92.2)
Removed third molar	b	**1649**	1.24	(1.01-1.52)	1633	(99.0)	0.97	(0.78-1.21)	1633	(99.0)	0.94	(0.74-1.18)	1633	(99.0)	0.93	(0.74-1.18)	1633	(99.0)
	c	**1740**	1.25	(1.02-1.52)	1720	(98.9)	0.97	(0.78-1.19)	1720	(98.9)	0.92	(0.73-1.16)	1719	(98.8)	0.93	(0.74-1.18)	1633	(93.9)
Restored teeth	b	**1638**	1.55	(1.12-2.14)	1519	(92.7)	1.34	(0.96-1.87)	1519	(92.7)	1.24	(0.87-1.78)	1519	(92.7)	1.23	(0.86-1.76)	1519	(92.7)
	c	**1740**	1.53	(1.12-2.10)	1611	(92.6)	1.32	(0.95-1.83)	1611	(92.6)	1.24	(0.88-1.76)	1610	(92.5)	1.23	(0.86-1.75)	1519	(87.3)
**Pharmacologically treated GDM-Diet-treated GDM**																		
Need for oral care	b	**939**	1.14	(0.80-1.61)	932	(99.3)	1.17	(0.82-1.66)	932	(99.3)	1.07	(0.75-1.54)	932	(99.3)	1.07	(0.74-1.53)	932	(99.3)
	c	**1001**	1.08	(0.77-1.52)	992	(99.1)	1.10	(0.78-1.55)	992	(99.1)	1.02	(0.72-1.44)	991	(99.0)	1.07	(0.74-1.53)	932	(93.1)
Gingival bleeding	b	**938**	1.12	(0.67-1.86)	928	(98.9)	1.21	(0.72-2.03)	928	(98.9)	1.19	(0.70-2.01)	928	(98.9)	1.13	(0.66-1.92)	928	(98.9)
	c	**1001**	1.05	(0.63-1.75)	989	(98.8)	1.14	(0.68-1.91)	989	(98.8)	1.13	(0.67-1.90)	988	(98.7)	1.13	(0.66-1.92)	928	(92.7)
Symptoms from the third molar	b	**939**	1.55	(1.11-2.14)	920	(98.0)	1.59	(1.14-2.22)	920	(98.0)	1.52	(1.09-2.13)	920	(98.0)	1.50	(1.07-2.11)	920	(98.0)
	c	**1001**	1.47	(1.07-2.02)	978	(97.7)	1.53	(1.10-2.11)	978	(97.7)	1.46	(1.05-2.02)	977	(97.6)	1.50	(1.07-2.11)	920	(91.9)
Removed third molar	b	**939**	1.20	(0.84-1.72)	929	(98.9)	0.98	(0.67-1.42)	929	(98.9)	0.96	(0.66-1.41)	929	(98.9)	0.96	(0.66-1.41)	929	(98.9)
	c	**1001**	1.11	(0.79-1.56)	988	(98.7)	0.90	(0.63-1.28)	988	(98.7)	0.88	(0.62-1.27)	987	(98.6)	0.96	(0.66-1.41)	929	(92.8)
Restored teeth	b	**932**	0.71	(0.41-1.22)	873	(93.7)	0.62	(0.35-1.08)	873	(93.7)	0.59	(0.33-1.03)	873	(93.7)	0.59	(0.33-1.03)	873	(93.7)
	c	**1001**	0.74	(0.44-1.25)	936	(93.5)	0.63	(0.37-1.07)	936	(93.5)	0.59	(0.34-1.01)	935	(93.4)	0.59	(0.33-1.03)	873	(87.2)

**Recurrent GDM-Control**																		
Need for oral care	b	**1108**	1.77	(1.29-2.44)	1097	(99.0)	1.91	(1.35-2.69)	1097	(99.0)	1.85	(1.27-2.70)	1097	(99.0)	1.85	(1.26-2.71)	1097	(99.0)
	c	**1168**	1.90	(1.40-2.58)	1156	(99.0)	2.07	(1.49-2.88)	1156	(99.0)	2.00	(1.39-2.88)	1156	(99.0)	1.85	(1.26-2.71)	1097	(93.9)
Gingival bleeding	b	**1107**	0.87	(0.51-1.49)	1098	(99.2)	1.04	(0.59-1.82)	1098	(99.2)	0.90	(0.49-1.67)	1098	(99.2)	0.89	(0.48-1.66)	1098	(99.2)
	c	**1168**	0.83	(0.50-1.40)	1158	(99.1)	1.02	(0.59-1.77)	1158	(99.1)	0.89	(0.49-1.63)	1158	(99.1)	0.88	(0.47-1.64)	1099	(94.1)
Symptoms from the third molar	b	**1108**	1.26	(0.93-1.71)	1073	(96.8)	1.31	(0.95-1.82)	1073	(96.8)	1.32	(0.92-1.89)	1073	(96.8)	1.25	(0.87-1.80)	1073	(96.8)
	c	**1168**	1.25	(0.93-1.68)	1128	(96.6)	1.28	(0.93-1.75)	1128	(96.6)	1.28	(0.91-1.82)	1128	(96.6)	1.25	(0.87-1.80)	1073	(91.9)
Removed third molar	b	**1108**	1.70	(1.22-2.39)	1096	(98.9)	1.14	(0.79-1.64)	1096	(98.9)	1.09	(0.73-1.62)	1096	(98.9)	1.11	(0.74-1.66)	1096	(98.9)
	c	**1168**	1.68	(1.21-2.32)	1155	(98.9)	1.09	(0.77-1.56)	1155	(98.9)	1.06	(0.72-1.55)	1155	(98.9)	1.11	(0.74-1.66)	1096	(93.8)
Restored teeth	b	**1101**	2.07	(1.33-3.22)	1014	(92.1)	1.63	(1.01-2.64)	1014	(92.1)	1.61	(0.95-2.74)	1014	(92.1)	1.58	(0.92-2.69)	1014	(92.1)
	c	**1168**	2.00	(1.30-3.08)	1073	(91.9)	1.54	(0.96-2.46)	1073	(91.9)	1.48	(0.88-2.50)	1073	(91.9)	1.58	(0.92-2.69)	1014	(86.8)
**First GDM-Control**																		
Need for oral care	b	**1645**	1.28	(1.03-1.59)	1633	(99.3)	1.45	(1.16-1.83)	1633	(99.3)	1.33	(1.04-1.69)	1633	(99.3)	1.30	(1.02-1.67)	1633	(99.3)
	c	**1732**	1.26	(1.01-1.55)	1719	(99.2)	1.43	(1.15-1.79)	1719	(99.2)	1.31	(1.04-1.67)	1718	(99.2)	1.30	(1.02-1.67)	1633	(94.3)
Gingival bleeding	b	**1645**	1.21	(0.88-1.66)	1627	(98.9)	1.30	(0.94-1.81)	1627	(98.9)	1.31	(0.92-1.86)	1627	(98.9)	1.28	(0.90-1.82)	1627	(98.9)
	c	**1732**	1.17	(0.86-1.60)	1713	(98.9)	1.26	(0.92-1.74)	1713	(98.9)	1.29	(0.91-1.81)	1712	(98.8)	1.28	(0.89-1.82)	1627	(93.9)
Symptoms from the third molar	b	**1645**	1.13	(0.92-1.38)	1598	(97.1)	1.14	(0.93-1.41)	1598	(97.1)	1.10	(0.88-1.38)	1598	(97.1)	1.06	(0.84-1.33)	1598	(97.1)
	c	**1732**	1.15	(0.94-1.39)	1680	(97.0)	1.16	(0.95-1.43)	1680	(97.0)	1.12	(0.90-1.39)	1679	(96.9)	1.06	(0.85-1.33)	1598	(92.3)
Removed third molar	b	**1645**	1.19	(0.97-1.47)	1629	(99.0)	0.92	(0.74-1.15)	1629	(99.0)	0.90	(0.71-1.14)	1629	(99.0)	0.90	(0.71-1.14)	1629	(99.0)
	c	**1732**	1.18	(0.97-1.44)	1712	(98.8)	0.91	(0.73-1.12)	1712	(98.8)	0.88	(0.70-1.10)	1711	(98.8)	0.9	(0.71-1.14)	1629	(94.1)
Restored teeth	b	**1635**	1.26	(0.90-1.77)	1521	(93.0)	1.11	(0.78-1.57)	1521	(93.0)	1.03	(0.71-1.50)	1521	(93.0)	1.02	(0.70-1.48)	1521	(93.0)
	c	**1732**	1.29	(0.93-1.78)	1610	(93.0)	1.14	(0.81-1.59)	1610	(93.0)	1.08	(0.75-1.54)	1609	(92.9)	1.02	(0.70-1.48)	1521	(87.8)
**Recurrent GDM-First GDM**																		
Need for oral care	b	**965**	1.39	(1.00-1.91)	958	(99.3)	1.37	(0.97-1.92)	958	(99.3)	1.30	(0.92-1.83)	958	(99.3)	1.31	(0.93-1.86)	958	(99.3)

	c	**1030**	1.52	(1.11-2.06)	1021	(99.1)	1.48	(1.07-2.06)	1021	(99.1)	1.41	(1.01-1.97)	1020	(99.0)	1.31	(0.93-1.86)	958	(93.0)
Gingival bleeding	b	**964**	0.73	(0.43-1.24)	953	(98.9)	0.81	(0.46-1.43)	953	(98.9)	0.80	(0.45-1.41)	953	(98.9)	0.80	(0.45-1.41)	953	(98.9)
	c	**1030**	0.71	(0.42-1.19)	1017	(98.7)	0.81	(0.47-1.41)	1017	(98.7)	0.81	(0.47-1.41)	1016	(98.6)	0.79	(0.45-1.41)	953	(92.5)
Symptoms from the third molar	b	**965**	1.12	(0.82-1.52)	943	(97.7)	1.17	(0.84-1.62)	943	(97.7)	1.12	(0.80-1.57)	943	(97.7)	1.15	(0.82-1.62)	943	(97.7)
	c	**1030**	1.09	(0.81-1.47)	1004	(97.5)	1.15	(0.83-1.58)	1004	(97.5)	1.11	(0.80-1.53)	1003	(97.4)	1.14	(0.82-1.60)	943	(91.6)
Removed third molar	b	**965**	1.43	(1.01-2.02)	955	(99.0)	1.19	(0.82-1.74)	955	(99.0)	1.19	(0.81-1.74)	955	(99.0)	1.21	(0.83-1.78)	955	(99.0)
	c	**1030**	1.42	(1.02-1.98)	1017	(98.7)	1.18	(0.82-1.69)	1017	(98.7)	1.18	(0.82-1.70)	1016	(98.6)	1.21	(0.83-1.78)	955	(92.7)
Restored teeth	b	**958**	1.64	(1.05-2.55)	895	(93.4)	1.47	(0.91-2.36)	895	(93.4)	1.43	(0.89-2.31)	895	(93.4)	1.47	(0.91-2.37)	895	(93.4)
	c	**1030**	1.55	(1.01-2.38)	959	(93.1)	1.29	(0.82-2.04)	959	(93.1)	1.26	(0.80-2.00)	958	(93.0)	1.47	(0.91-2.37)	895	(86.9)

Model 1 included maternal age and parity, Model 2 included Model 1 + pre-pregnancy BMI and Model 3 included Model 2 + smoking during pregnancy, education and history of asthma and insomnia and/or mental disorders. Chronic hypertension, gestational hypertension and pre-eclampsia were included in Model 3 when gingival bleeding was analyzed. Hyperemesis was included in Model 3 when analyzing the number of restored teeth.

[a] n included to the model

[b] No missing variables in analyses

[c] Missing variables includes in analyses

GDM, gestational diabetes mellitus; OR, odds ratio; aOR, adjusted odds ratio; CI, confidence interval
